# Wearable Technology and Chronic Illness: Balancing Justice and Care Ethics

**DOI:** 10.7759/cureus.73686

**Published:** 2024-11-14

**Authors:** Zoe Lewczak, Maika Mitchell

**Affiliations:** 1 Bioethics, Harvard Medical School, Boston, USA; 2 Oncology, University of Maryland, Adelphi, USA

**Keywords:** benefits of wearable technology, care ethics, chronic illness, data security, ethical implications of wearable technology, healthcare technology, informed consent, medical ethics, remote patient monitoring, wearable health devices

## Abstract

The management of chronic diseases has been revolutionized by the advent of wearable health technology. These devices provide personalized and real-time health data to patients. The problem that this technology is most frequently used to address is obesity and its consequences, heart disease, and certain cancers. Numerous studies have shown a correlation between being at a healthy weight and having a lower risk of developing these conditions. Yet, many smart health devices in the wearable technology sector are only, and most of the time, within the context of weight management or obesity. The major research question directing this exploration is as follows: How can the use of wearable technologies be effective in improving the management and quality of life of people with chronic health problems? The question carries with it another that is almost as important: Does the use of these devices move us toward a more moral and just healthcare system, or does it unfairly advantage some groups of patients over others? This research will focus on three specific types of wearable devices, chosen as representative case studies. They are (1) the smartwatch, a more recent advancement in wearable technology that monitors the user's heart rate and physical activity; (2) the continuous glucose monitor (CGM), which presents real-time glucose levels for the user; and (3) the continuous positive airway pressure (CPAP) device, also called a respirator, worn during sleep by individuals diagnosed with sleep apnea. Each of these devices has the potential to not only revolutionize the management of chronic health conditions but also raise some important questions about the ethics of doing so.

## Introduction and background

Wearable health devices have the potential to transform healthcare, offering innumerable benefits for individuals with chronic illnesses. From better diagnostics, more effective treatments, and proactive engagement in maintaining health, this technology can improve health and quality of life. To make these almost utopian advantages real, we must consider the interplay of ethical tensions.

Chronic illnesses devastatingly affect people across the world, and according to the World Health Organization (WHO), "chronic diseases cause 71% of all deaths" [1]. The rising number of people with chronic illnesses also leads to a never-seen-before financial burden on healthcare systems; therefore, more effective and efficient management of chronic care is needed [1]. Traditional methods of managing these conditions often rely on periodic check-ups and reactive interventions, which can be inefficient and delay timely care. Wearable technology offers a promising solution by enabling continuous monitoring of vital signs and other relevant health metrics. This real-time data can empower both patients and healthcare providers to make informed decisions and take proactive steps to prevent disease progression and manage symptoms [2].

While there are many potential benefits of wearable technology, it is crucial to acknowledge the ethical considerations. The devices we can wear, including smartwatches, fitness monitors, and medical devices, represent an emerging source of big data collection. They capture a wealth of information about our lives and translate that data into insights that are both meaningful and, in many cases, prophetic. As these devices collect sensitive information, safeguards must be implemented to protect individuals' privacy, prevent unauthorized access, and ensure proper care. Equally as important is ensuring that wearable technology is accessible to all, regardless of socioeconomic status or geographic location, to avoid exacerbating existing health disparities [3].

The relationship of these devices to health and medicine is unambiguous and is undergirded by theoretical frameworks such as care ethics and justice. These frameworks support the idea that all the forms of data we can now collect about our lives should be used to help us in ways that are integrated, ethical, and more focused on the medicine of the future [3]. Care ethics emphasizes compassion, empathy, and responsiveness to individual needs, while justice ethics focuses on fairness, equity, and the distribution of benefits and burdens [4]. Just as a gardener nurtures a plant, providing it with the right conditions to flourish, so too must we carefully cultivate the use of wearable technology. We must ensure that this technology is accessible to all, regardless of socioeconomic status, and that it is used in a way that promotes the well-being of individuals and society as a whole. By understanding the complexities of this emerging field, we can harness the power of wearable technology to improve healthcare while safeguarding individuals and promoting equitable access.

## Review

The latest scholarly work emphasizes that the use of wearable technology is steadily increasing in the regulation of chronic diseases. These devices offer an unparalleled opportunity to monitor health metrics continuously. With the data gained from using wearable technology, healthcare professionals can provide better interventions and an even more personalized form of medicine compared to what is available today. Indeed, we are in an exciting era in which improved adherence to treatment protocols and better self-management seem possible as a direct result of using wearable technology [5].

Wearable technology includes a variety of devices, each with its own specific purpose. Fitness trackers, like Fitbit and Garmin, are not just glorified pedometers. They keep an eye on the heart, too. Many of these popular devices can track not only how much you move but also your heart rate and whether you're going to be dreaming or not. They can even give you a thumbs-up when it seems like you might be on your way to having a healthy day; unfortunately, they can't always help you with that [6].

By far, the most popular of these are smartwatches, especially the Apple Watch. These offer many of the same features and benefits as fitness trackers; however, they're not just for workouts. These can keep you fit, yes, but they can also help keep you alive if you have certain medical conditions to manage because they offer features you won't find in a lot of other health-related gadgets [6]. Promising devices that can be worn have emerged as a management tool for an array of ongoing illnesses. These potentially powerful management tools can help several chronic conditions, among them: diabetes, cardiovascular diseases (e.g., hypertension and heart failure), ongoing respiratory conditions (e.g., sleep apnea and chronic obstructive pulmonary disease (COPD)), obesity, ongoing mental health issues (like anxiety and depression), and disorders of the musculoskeletal system (e.g., arthritis) [7-9].

Wearable technologies offer many advantages for managing chronic diseases, including real-time monitoring, data-driven decisions, and leveraging patient and provider agency. Patients with chronic diseases can use wearable technology to constantly track their symptoms and biometric data, providing useful and immediate information about their current health status [9]. This technology allows for eHealth in a new modality, with the capacity for true immediacy in chronic illness management. Healthcare providers and patients can now use the collected data not just to act upon problems when they arise but also to anticipate "events" (e.g., the onset of a flare-up) and then tailor treatments accordingly. Using real-time data in conjunction with their usual practices, healthcare providers can now make more informed decisions. When the provider and the pace of life for the patient are considered, the wearables give greater context [10-12].

While wearable technology offers numerous potential benefits, it also raises significant ethical considerations. A major concern is data privacy and security. Since health data is among the most sensitive personal information, its security must be paramount. Wearable devices collect vast amounts of personal data, which could be compromised through malicious hacking or simple negligence, potentially falling into the wrong hands. Device security is another critical issue. Manufacturers must implement robust security measures to protect both the devices themselves and the data they collect from cyberattacks. The sheer volume of personal data collected by these devices raises questions about their privacy and potential misuse. It is unclear whether this data is any less private than that collected by smartphones [13-14]. Accessibility is another important factor. To realize the full potential of wearable technology in healthcare, it must be accessible to all patients [15]. If not, it risks exacerbating existing health disparities rather than mitigating them. The high cost of many wearable devices, such as the popular Apple Watch, can be a significant barrier for many individuals [16]. Finally, the psychological impact of a diagnosis and the need for ongoing treatment can influence overall well-being, making it crucial to strike a balance between leveraging technology to improve health and avoiding excessive reliance on it. Overreliance on wearable health devices can have negative psychological consequences, such as heightened health anxiety and a disconnect from one's own body [17].

To delve deeper, we will examine three case studies of wearable devices: smartwatches, continuous glucose monitors (CGMs), and continuous positive airway pressure (CPAP) devices. By analyzing these devices, we can better understand the potential benefits and the ethical considerations surrounding their application to provide care that is both safe and equitable. These devices were selected to represent a range of wearable technologies, each addressing distinct chronic illnesses. Smartwatches are employed for general health monitoring, CGMs are specifically designed for diabetes management, and CPAP devices are commonly used to treat sleep apnea [3,17,18]. By analyzing these distinct applications, we can gain a comprehensive understanding of the potential benefits and ethical considerations surrounding wearable technology in various healthcare contexts. While these devices offer significant potential for improving health outcomes, it is crucial to address the ethical challenges associated with their use, including data privacy, equity of access, and psychological impact.

Case 1: smartwatches

Beyond the functionalities of timekeeping, smartwatches have emerged as ubiquitous technological companions. These wearable devices are equipped with advanced medical-grade sensors and connectivity features, with the potential to reshape chronic disease management. Cardiovascular diseases, such as hypertension, heart failure, and a variety of arrhythmias, are prime targets for smartwatch applications. Through the tracking of heart rate, blood oxygen levels, and electrocardiogram data, these devices can enable early detection of irregularities and timely medical intervention outside the traditional clinical setting [18].

In addition to personalized health management, smartwatches have the potential to facilitate remote patient monitoring, allowing healthcare providers to track patients' health status from their own homes, reducing the need for frequent in-person visits [19]. This level of autonomy is particularly beneficial for individuals with chronic conditions who require regular monitoring; the smartwatch data can provide valuable insights for clinicians, allowing for diagnosis, treatment, and assessment of treatment efficacy of treatments at any time [20]. However, it’s important to recognize the potential for overreliance on these devices, which can have detrimental effects like health anxiety. There is potential to create a culture of constant self-monitoring where individuals become bombarded with data and feel pressure to optimize every aspect of their lives - from obsessing over numbers like step count or fixating on sleep scores. The constant monitoring of biometrics can lead to a preoccupation with minor fluctuations and a heightened sense of vulnerability [19]. Striking a balance between data-driven insights and a holistic approach to health is a crucial component of care.

These devices collect sensitive health information, which is vulnerable to breaches, so a major concern is data privacy and security. This is an area where the legal system has not caught up to the medico-technological landscape and is lacking in consumer protections. Initially, many of these wearables were created around the fitness industry, so there were few regulatory standards required. However, in 2021, the United States Food and Drug Administration approved the Apple Watch Series 6 electrocardiogram app for detecting atrial fibrillation. Yet, many of the commercially available smartwatches are not yet classified as medical devices. Although the software in many smartwatches aligns with the accuracy of medical devices, they cannot support clinical decision-making without legal regulation [21]. This regulatory gap necessitates careful consideration when using wearable data for clinical decision-making.

Case 2: continuous glucose monitors

Next, it’s important to look at CGMs, which have significantly advanced diabetes management by providing real-time, continuous glucose monitoring. Unlike traditional finger-prick testing, offering intermittent snapshots of blood glucose levels, CGMs utilize a sensor inserted under the skin to measure interstitial glucose levels. This technology enables individuals with diabetes to gain a more comprehensive understanding of their glucose patterns throughout the day and night [17].

By continuously tracking glucose levels, CGMs can help individuals with diabetes achieve better glycemic control, reduce the risk of hypoglycemia, and enhance overall diabetes management, all of which are life-threatening side effects of changes to glucose levels. By providing real-time data, CGMs can detect and alert users to impending hypoglycemia, allowing them to take timely corrective actions. Studies have shown that CGM use can lead to improved HbA1c levels, indicating better long-term blood sugar control. Additionally, CGMs empower individuals to make informed decisions about diet, exercise, and insulin dosage, leading to improved self-management [17].

There are several challenges associated with the use of CGMs. Much like the data privacy and security concerns with smartwatches, these devices collect sensitive health information, which can be subject to breaches [21]. Another concerning feature is that the high cost of CGMs and associated sensor replacements can limit access for many individuals, particularly in low-income populations. Furthermore, ensuring the accuracy and reliability of CGM sensors is crucial to prevent misinterpretation of data and potential adverse health consequences [17].

Case 3: continuous positive airway pressure

Another wearable health technology is the CPAP device, a non-invasive treatment delivering a constant stream of pressurized air through a mask worn over the nose or nose and mouth. Primarily used to treat obstructive sleep apnea (OSA), CPAP therapy has also found applications in managing other respiratory disorders, such as premature infant respiratory distress syndrome and COPD [22].

By maintaining open airways and improving oxygenation, CPAP therapy offers numerous benefits, including improved sleep quality, reduced daytime sleepiness, improved cardiovascular health, and enhanced quality of life. For individuals with OSA, this therapy can significantly reduce the frequency and severity of apneic events, leading to better sleep quality and reduced daytime fatigue. In premature infants, CPAP therapy can help stabilize breathing, improve oxygenation, and reduce the risk of lung damage. For individuals with COPD, CPAP therapy can alleviate symptoms, improve exercise tolerance, and enhance overall quality of life [22].

However, the widespread use of CPAP therapy is hindered by several challenges. Much like the cost concerns around CGMs, the high cost of CPAP devices and the ongoing need for supplies, such as masks and filters, can limit access for many individuals. Even with access, some individuals have difficulty or discomfort using CPAP therapy, so if it’s sitting on the nightstand and not being used, it’s not doing much good. A CPAP device, like any medical technology, is only as effective as the patient's adherence to therapy. Some patients report discomfort or difficulty adjusting to the therapy. Factors such as mask fit, side effects like skin irritation or claustrophobia, and complex machine settings can further hinder adherence. Not only that, the psychological impact of a diagnosis and the need for ongoing treatment can influence patient motivation. Addressing these challenges, including ensuring a comfortable fit, managing side effects, and providing clear instructions, is crucial for long-term adherence and the success of CPAP therapy in managing respiratory disorders [22].

Limitations

While wearable health technology offers significant potential for improving health outcomes, it is essential to recognize its technological limitations. Issues such as data accuracy, battery life, and user interface design can hinder the effectiveness and widespread adoption of these devices. For instance, inaccuracies in sensor data can lead to misinterpretations and incorrect treatment decisions [19]. Limited battery life can restrict continuous monitoring, and complex user interfaces can discourage adherence to prescribed protocols [23]. To fully realize the benefits of wearable technology, it is crucial to prioritize research and development to address these limitations and ensure that these devices are user-friendly, reliable, and accessible to all. By doing so, we can unlock the potential of wearable technology to improve health outcomes while mitigating potential risks and ethical concerns.

Discussion

One pressing ethical concern with wearable health technology is data privacy. These devices collect vast amounts of sensitive personal information, raising questions about the extent to which individuals are willing to relinquish their privacy for health benefits. It's akin to inviting a guest into your home who records every detail of your life. Then, this guest shares your intimate information with outside parties, like app developers or data analytics firms. While you may not be aware of the extent of your data sharing, there lies the inherent potential for misuse and mistrust, particularly among those skeptical of the technology. Additionally, ensuring equitable access to wearable technology is crucial. Socioeconomic disparities, technological literacy, and internet connectivity can hinder adoption, potentially exacerbating existing health disparities. A justice-based approach can address both of these concerns by prioritizing data privacy and equitable access. This approach would involve implementing robust data protection measures, ensuring transparency in data collection and usage, and promoting the development of affordable and accessible wearable devices. By prioritizing justice, we can ensure that the benefits of wearable technology are distributed fairly and that individuals' privacy rights are protected [4].

Care ethics, on the other hand, emphasizes human connections through empathy and compassion, offering a valuable counterbalance to the more theoretical justice-based approach. Healthcare professionals play a crucial role in navigating the ethical landscape of wearable health technologies. These devices can serve as valuable tools for collecting and monitoring patient data, but it's essential that healthcare providers interpret this data accurately and provide compassionate, personalized guidance. Individuals should not be reduced to mere data points; rather, they should be treated with empathy [20,4]. Striking a balance between data-driven insights and a holistic approach to health is crucial to avoid overreliance on technology and its potential negative psychological consequences, such as diminished motivation for treatment and health anxiety.

The future of wearable health devices

To fully recognize the potential of wearable technology in chronic disease management, we must address the ethical and practical challenges while embracing the paradigm shift from a paternalistic model of care to one centered on patient empowerment [23]. Rigorous empirical research is necessary to evaluate its impact on health outcomes, quality of life, and healthcare costs [21]. Simultaneously, a collaborative effort involving healthcare professionals, technology companies, and policymakers is essential to developing comprehensive justice frameworks that prioritize data privacy, security, and equitable access [4]. By establishing clear guidelines for device safety, interoperability, and affordability, we can ensure that wearable technologies are used responsibly and effectively [21]. Lastly, through human-centered design, these technologies can empower individuals to take control of their health while also supporting healthcare professionals in providing more personalized and effective care.

## Conclusions

As wearable health devices continue to evolve, it’s important to engage in an ethical dialogue, addressing pressing issues like privacy and security, accessibility, and psychological impacts. While data-driven technology offers valuable potential, we must ensure that it complements, rather than replace, the essential human connection between patients and healthcare providers. By acknowledging justice and the necessary human elements, wearable health technology ought to enhance the doctor-patient relationship, not replace it.
